# Plague Epidemics and Lice, Democratic Republic of the Congo

**DOI:** 10.3201/eid1903.121542

**Published:** 2013-03

**Authors:** Renaud Piarroux, Aaron Aruna Abedi, Jean-Christophe Shako, Benoit Kebela, Stomy Karhemere, Georges Diatta, Bernard Davoust, Didier Raoult, Michel Drancourt

**Affiliations:** Author affiliations: Aix-Marseille University, Marseilles, France (R. Piarroux, B. Davoust, D. Raoult, M. Drancourt);; Ministry of Health, Kinshasa, Democratic Republic of the Congo (A. Aruna Abedi, B. Kebela);; Plague Reference Laboratory, Bunia, Democratic Republic of the Congo (J.C. Shako);; National Institute of Biomedical Research, Kinshasa (S. Karhemere);; Institute of Research for the Development, Dakar, Senegal (G. Diatta)

**To the Editor:** Plague, a zoonotic disease caused by the gram-negative bacterium *Yersinia pestis*, is transmitted to humans by the bites of infected fleas (such as *Xenopsylla cheopis*), scratches from infected animals, and inhalation of aerosols or consumption of food contaminated with *Y. pestis* (*1*). Decades ago, Blanc and Baltazard proposed that human-to-human transmission of *Y. pestis* could be mediated by human ectoparasites, such as the human body louse (*2*). This hypothesis was further supported by experimental data from animal models (*3*).

To further test this hypothesis among humans, we conducted a field assessment in April 2010, in which we collected body and head lice from persons living in a highly plague-endemic area near the Rethy Health District, Province Orientale, Democratic Republic of the Congo. This health district has 157,000 inhabitants, and during 2004–2009 it had more suspected plague cases (1,624 cases of suspected plague, 39 deaths) than any other health district in the Democratic Republic of the Congo. In April 2010, we visited the dwellings of 10 patients for whom suspected cases of plague had been diagnosed during January–April 2010. All patients had symptoms typical of bubonic plague, and their illnesses were reported as suspected bubonic plague. However, because of the lack of laboratory facilities in Rethy, none of these diagnoses could be microbiologically confirmed. 

A total of 154 body lice and 35 head lice were collected from clothes and hair of persons living in or near the patients’ dwellings. Body lice were preserved in ethanol before being sent to the laboratory. Total DNA was extracted by using an EZ1 automated extractor (QIAGEN, Courtaboeuf, France) and subjected to parallel real-time PCRs selective for the *Y. pesti*s *pla* gene, the *Rickettsia prowazekii omp*B gene, a *Borrelia recurrentis* noncoding genomic fragment, and the *Bartonella quintana* internal transcribed spacer. *B. quintana* PCR primers and probe have been shown to be specific for *B. quintana* (*4*). Primers and probe sequences and experimental conditions have been reported (*4*). Negative controls contained PCR buffer without DNA, as described (*4*). Any amplification with a cycle threshold (C_t_) <40 was regarded as positive.

Negative controls remained negative in all PCR-based experiments, which were not prone to in-laboratory contamination, and all samples were negative for *R. prowazekii* and *B. recurrentis*. Conversely, *B. quintana* was detected in 50 (32.5%) of the 154 body lice (C_t_ 18.62–38.45) and 6 (17.1%) of the 35 head lice (C_t_ 29.48–38.68). The *Y. pestis pla* gene was detected in 1 head louse (C_t_ 38), which was negative for the other pathogens, and in 2 body lice (C_t_ 37.36 and 36.97, respectively), which were positive for *B. quintana*.

*B. quintana* has been detected in head louse specimens collected in Ethiopia and Senegal (*5*–*7*) and in body louse specimens collected in Burundi, Rwanda, Zimbabwe (*8*), and Ethiopia (*5*); we add Democratic Republic of the Congo to the list. Body lice are acknowledged vectors for human-to-human transmission of *B. quintana* (*9*). Detection of *Y. pestis* in head and body lice has been reported (*2*). Detailed observations in south Morocco showed that body lice collected from blood culture–negative bubonic plague patients were negative for *Y. pestis*, whereas body lice collected from septicemic patients were positive according to guinea pig inoculation results (*2*). Further experiments in a rabbit experimental model demonstrated the possibility of direct louse-bite transmission of *Y. pestis* (*3*). A recent search for *Y. pestis* in head lice in Ethiopia found none (*5*). 

Our detection of *B. quintana* and the plague agent *Y. pestis* in modern head and body lice is similar to findings of a paleomicrobiological investigation at a medieval plague site near Paris (*10*). There, high-throughput real-time PCR investigation of dental pulp collected from 14 teeth from 5 skeletons detected *B. quintana* DNA in teeth from 3 skeletons and *Y. pestis* DNA in teeth from 2, including 1 with co-infection. Altogether, these data suggest that transmission of *B. quintana* and *Y. pestis* has been ongoing for centuries in populations in which louse infestation is prevalent. This finding indicates that lice might play a role in transmission of *Y. pestis* and that preventing and controlling louse infestations might help limit the extension of plague epidemics in louse-infested populations.
